# Early structural valve deterioration following transcatheter aortic valve implantation in a patient with Scheie syndrome: a case report

**DOI:** 10.1186/s44215-024-00170-6

**Published:** 2024-10-21

**Authors:** Yusuke Yanagino, Satoshi Kainuma, Koichi Toda, Ai Kawamura, Takuji Kawamura, Daisuke Yoshioka, Masaki Taira, Kazuo Shimamura, Shigeru Miyagawa

**Affiliations:** Department of Cardiovascular Surgery, Osaka University Graduate School of Medicine, Suita, Osaka 565-0871 Japan

**Keywords:** Scheie syndrome, Mucopolysaccharidosis type I, Structural valve deterioration, Transcatheter aortic valve implantation, Glycosaminoglycans, Coronary artery bypass grafting

## Abstract

**Background:**

Scheie syndrome, an attenuated subtype of mucopolysaccharidosis type I, is a rare storage disease that causes progressive glycosaminoglycans (GAGs) accumulation. Cardiovascular disorders determine the prognosis, and cardiac valve abnormalities are the most common cause. The patients are usually young so mechanical valve replacement is often performed, but because of the features of this disease, the surgical treatment is very risky. Recently, transcatheter aortic valve implantation (TAVI) has been reported as an alternative choice for aortic stenosis, but optimal choice is still unclear. Here, we introduce a patient that underwent TAVI and refer to the histological finding of a biological valve extracted in relation to GAGs accumulation.

**Case presentation:**

A 54-year-old woman with Scheie syndrome underwent valve surgeries three times throughout her whole life. At age 41, she received a mitral valve replacement with a mechanical valve for mitral stenosis. She promptly developed severe diastolic dysfunction and low output syndrome after the release of aortic clamping, thus requiring temporary mechanical circulatory support^4^. At age 51, she suffered from heart failure due to severe aortic stenosis and underwent TAVI because conventional aortic valve replacement (AVR) was deemed too risky. Three years later, her heart failure relapsed, and an echocardiogram unexpectedly revealed thickened bioprosthetic valve leaflets and a significant pressure gradient across the valve, consistent with early structural valve deterioration. AVR was performed via median sternotomy with a mechanical valve. As with the first operation, she presented refractory heart failure requiring mechanical circulatory support and was meticulously managed. However, she steadily showed worsening of multiple organ systems and died 9 days after the operation. Pathological autopsy and histological examination revealed accumulation of tissue with GAGs on the leaflets of the bioprosthetic valve of TAVI, which may have been the cause of early structural valve deterioration.

**Conclusion:**

For patients with Scheie syndrome, a biological valve can be compromised by the accumulation of GAGs, thereby causing early SVD. These findings may support valve selection for these high-risk patients.

## Background

Scheie syndrome, attenuated subtype of mucopolysaccharidosis type I (MPS-I), is the rare storage disease which causes an accumulation of glycosaminoglycans (GAGs) in multisystems, leading to cardiac valve abnormalities [1, 2]. Mechanical valve replacement is performed since the patients are generally young, but because of the features of this disease, the conventional surgical treatment is too risky. Recently, transcatheter aortic valve implantation (TAVI) has also been reported [3], but optimal choice is still unclear. We report an early structural valve deterioration (SVD) in a patient with Scheie syndrome 3 years after TAVI and propose its potential mechanism based on her autopsy findings.

## Case presentation

The patient was a 54-year-old woman. She was diagnosed with Scheie syndrome at age 8 and underwent an enzyme replacement therapy. Her valve abnormalities developed gradually; at age 41, she received mitral valve replacement with a 20-mm ATS mechanical valve (ATS Medical, Minneapolis, MN, USA) for mitral stenosis, after which she developed severe diastolic dysfunction and low output syndrome requiring mechanical circulatory supports with intra-aortic balloon pumping and extracorporeal membranous oxygenation for 3 days. At age 51, she suffered from heart failure due to severe aortic stenosis. Based on our experience from the first surgery, we opted for TAVI due to the high risk of surgical aortic valve replacement (AVR). Computed tomography showed a very small sino-tubular junction and sinus of Valsalva, making a self-expandable valve unsuitable. Additionally, due to 50–75% stenosis in the right coronary artery, future percutaneous coronary intervention may be necessary, so we chose a balloon-expandable valve. The aortic annulus was 280 mm^2^, and a 20-mm SAPIEN XT valve (Edwards Lifesciences, Irvine, CA, USA) was implanted in nominal volume. Intraoperative pressure measurements showed the mean pressure gradient of 0 mmHg. Postoperative transthoracic echocardiography showed mean pressure gradient of 16 mmHg and aortic valve area of 0.84 cm^2^ without any perivalvular leakage. The symptoms disappeared after the surgery, but 1 year later, the mean pressure gradient increased to 25 mmHg, and after 3 years, it rose to 30 mmHg. The patient developed exertional dyspnea and was diagnosed with recurrent heart failure, resulting in hospitalization.

**Fig. 1:**
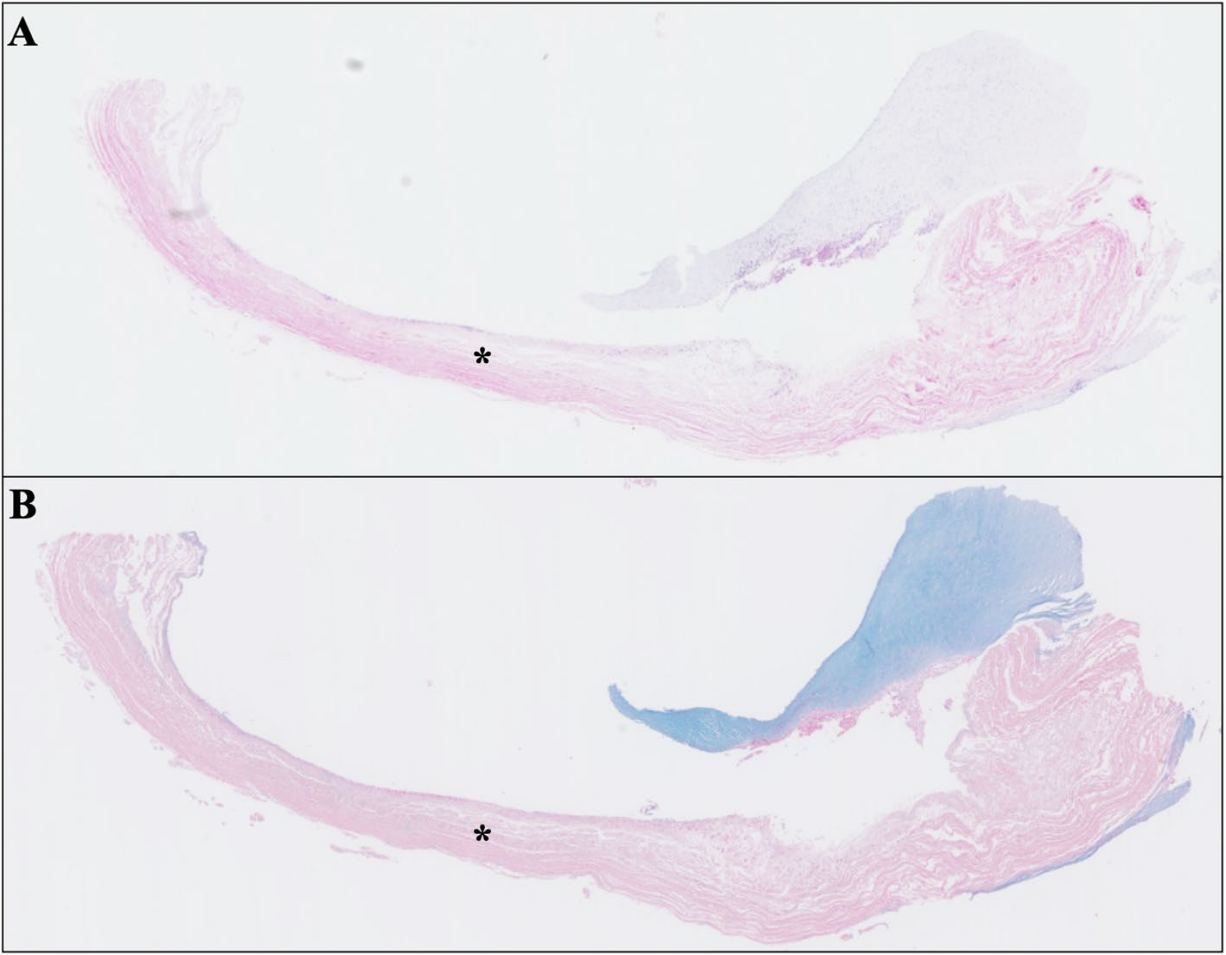
Prosthetic valve leaflet. Microscopic finding of the bioprosthetic aortic valve leaflets (asterisk) stained with hematoxylin–eosin (**A**) and Alcian Blue (**B**). Tissue stained with Alcian Blue was on the prosthetic valve leaflet indicated accumulation of acid mucopolysaccharides

**Fig. 2:**
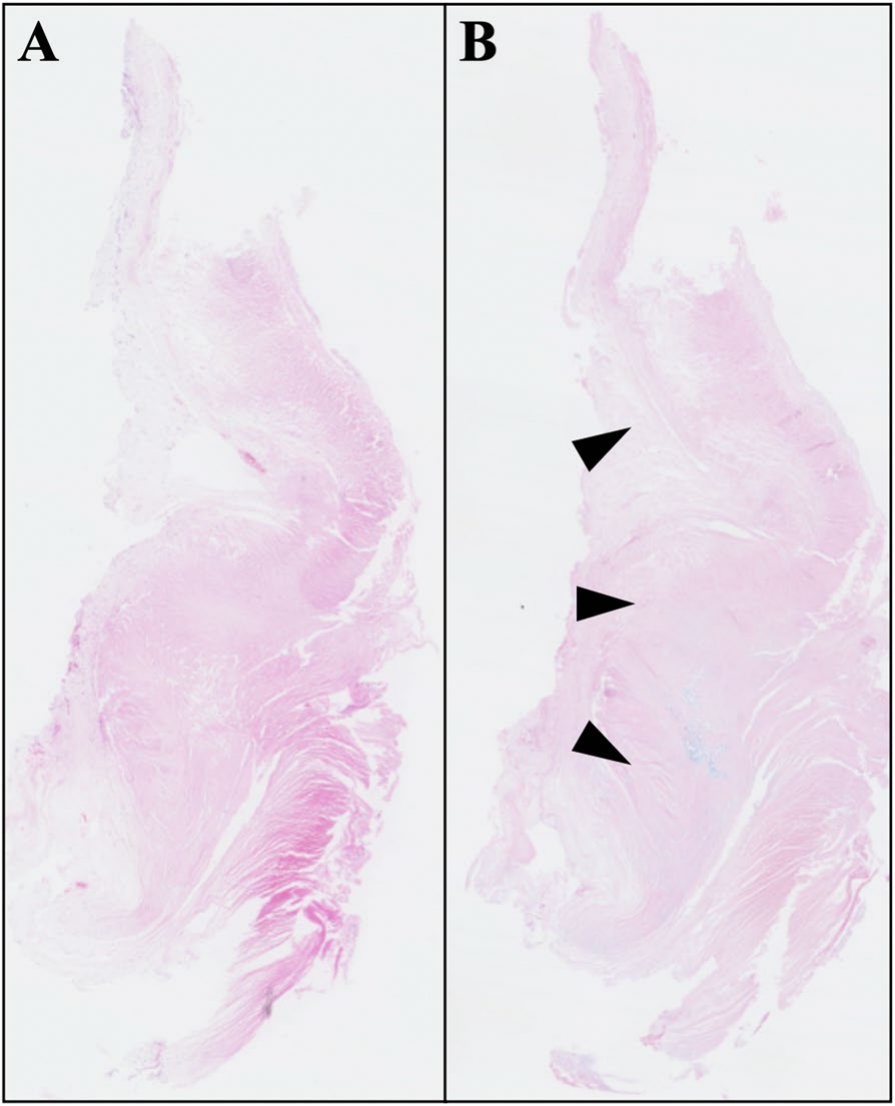
Native aortic valve leaflet. Microscopic finding of the native aortic leaflet stained with hematoxylin–eosin (**A**) and Alcian Blue (**B**). **B** Accumulation of mucopolysaccharides on the leaflet (black triangles)

**Fig. 3:**
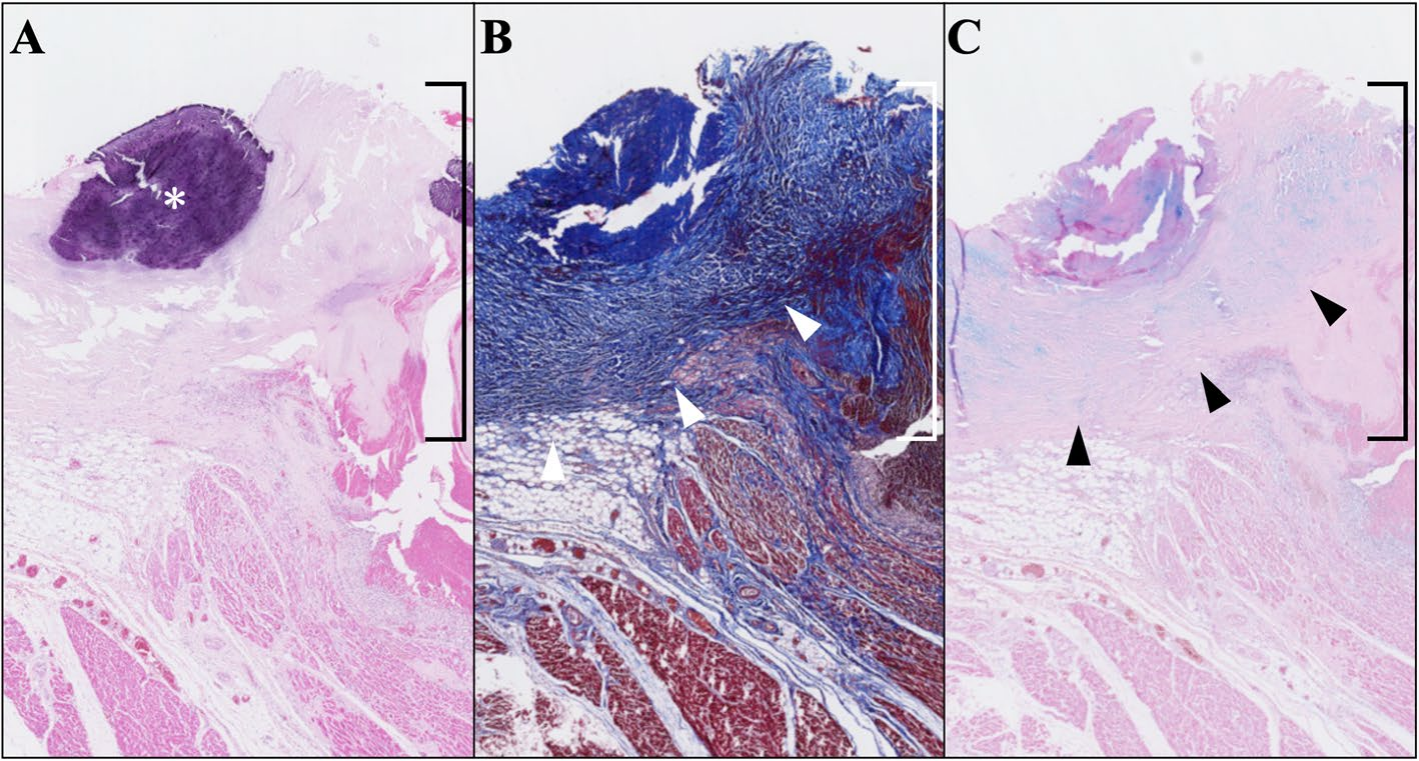
Native aortic annulus. Microscopic finding of the native aortic annulus (the area indicated by a right bracket) stained with hematoxylin–eosin (**A**), Masson trichrome (**B**), and Alcian Blue (**C**). There was cartilage-like change (**A**, asterisk), hyperplasia of collagenous tissue (**B**, white triangles), and accumulation of acid mucopolysaccharides (**C**, black triangles)

**Fig. 4:**
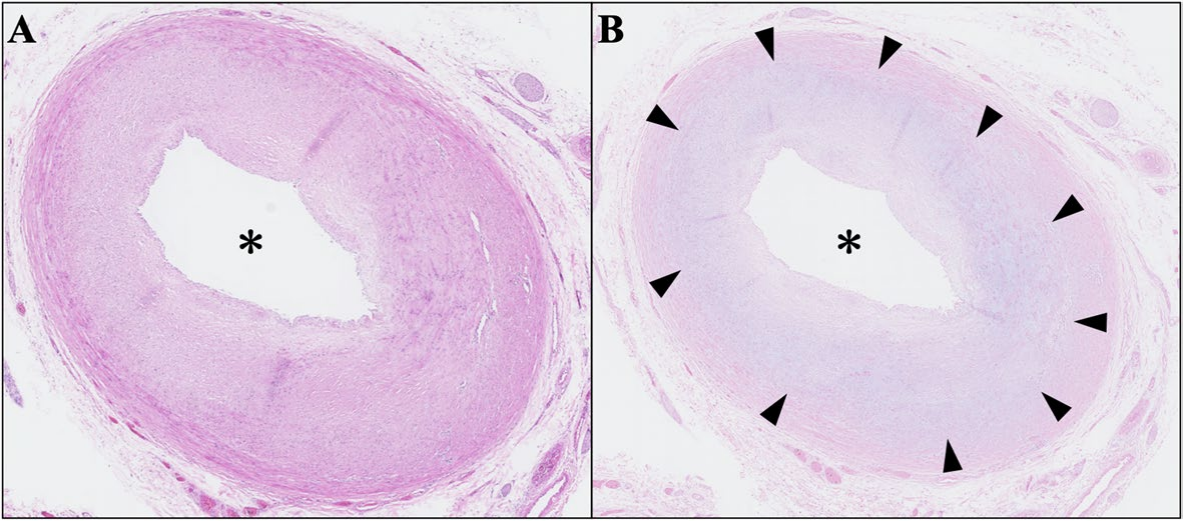
Left main trunk of the left coronary artery. Photomicrographs of the left main trunk of the coronary artery stained with hematoxylin–eosin (**A**) and Alcian Blue (**B**). Mucopolysaccharides were deposited in the intima (black triangles). Asterisks indicate the lumen of the coronary artery

**Fig. 5:**
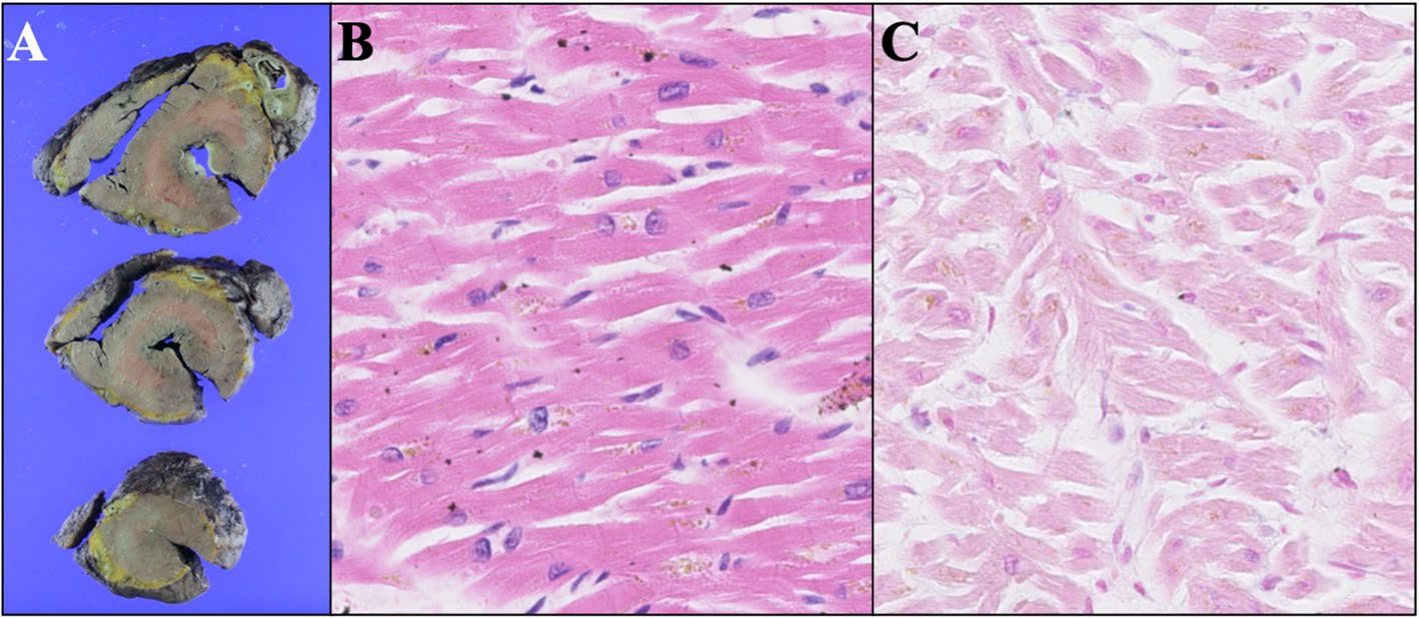
Left ventricle. Macroscopic specimen of the thickened left ventricle. **A** Photomicrographs of the left ventricle wall stained with hematoxylin–eosin (**B**) and Alcian Blue (**C**). Mucopolysaccharides were not deposited in the left ventricular wall

She was of average intelligence, and her height and weight were 146.5 cm and 37.5 kg, respectively. She had a short neck and a macroglossia, which are typical features of Scheie syndrome. Transthoracic echocardiography demonstrated prosthetic aortic valve deterioration as moderate-severe aortic stenosis shown by mean pressure gradient across the valve of 30 mmHg, peak flow velocity of 3.66 m/s, aortic valve area of 0.64 cm^2^, and mild-moderate perivalvular leakage. The left ventricular ejection fraction was preserved at 71%. We also suspected leaflet thrombosis on TAVI valve; however, neither preoperative contrasted CT scan nor transesophageal echocardiography clearly identified the image of the thrombus on prosthetic valve leaflets. Coronary angiography revealed 90% stenosis at the right coronary artery ostium. Although the patient’s mean pressure gradient was not extremely high and cardiac function was preserved, the patient suffered from heart failure symptoms, along with worsening pressure gradients and a decrease in valve area. Thrombus on the valve was ruled out. We also considered performing TAVI again; however, it was not covered by health insurance in Japan at that time. Additionally, the presence of moderate paravalvular leakage made this option less ideal. Therefore, after discussion by the heart team, it was concluded that surgical intervention was the only option to improve the symptoms. Then, the patient received coronary artery bypass grafting (CABG) and AVR with a 16-mm ATS AP360 (ATS Medical, Minneapolis, MN, USA) mechanical valve and augmentation of the noncoronary sinus of Valsalva with a bovine pericardial patch. Intraoperative findings showed no thrombus formation on the valve leaflets, which were thickened, consistent with SVD. She suffered from intraoperative refractory heart failure and severe pulmonary hypertension. Transesophageal echocardiography showed normal left ventricular contraction, leading us to diagnose left ventricular diastolic dysfunction. Mechanical circulatory support was required, similar to her first operation [4]. She could not be weaned from the mechanical circulatory support and died 9 days postoperatively. Pathological autopsy and histological examination revealed that GAGs were accumulated on the prosthetic (Fig. 1) and the native aortic valves and annulus. No thrombus formation was observed on the valve leaflets (Figs. 2 and 3). Hyperplasia of collagenous fibers and cartilage-like changes also occurred in the native aortic anulus (Fig. 3). The intima of the right coronary artery, left coronary trunk, and left anterior descending artery was thickened, and GAGs were also deposited in the arterial walls (Fig. 4); however, we did not find any GAG deposits or evidence of myocardial infarction in the left ventricular wall (Fig. 5).

## Discussion and conclusions

Scheie syndrome, an attenuated subtype of MPS-I, is a rare lysosomal storage disorder with a prevalence of 1/500,000. It is characterized by a deficiency in the enzymatic activity of alpha-L-iduronidase leading to the progressive accumulation of GAGs in the bones, cartilage, eyes, and cardiovascular system [1] along with valvular abnormalities primarily affecting the left-sided valves [2]. Scheie syndrome is rare: only 10 cases have been reported between 1989 and 2015, in which 11 valve surgeries were performed (Table 1). The patients received mechanical valve implantation in the aortic and/or mitral valve position except for one case which underwent TAVI [3], because these patients were relatively young when they underwent valve surgery, with a mean age of 41. Additionally, the annuli of the aortic and mitral valves are often too small to implant a biological prosthetic valve. Aortic root enlargement is sometimes required to implant of the mechanical valve in an aortic position [5]. The durability of both biological and TAVI valves remains unknown in patients with Scheie syndrome. In our case, TAVI was considered the safest option as the patient developed low output, severe pulmonary hypertension and diastolic dysfunction, thereby requiring mechanical circulatory support for several days after the first operation.

**Table 1:** Previous reports of valve surgery in patients with Scheie syndrome

Authors	Age	Gender	Valve surgery	Aortic valve prosthesis	Mitral valve prosthesis	Concomitant CABG	Postoperative device support	Outcome
Butman S. M. et al.	42	Female	AVR + MVR	20-mm Medtronic Hall	25-mm St. Jude	-	Not required	Alive
Masuda H. et al.	62	Male	AVR	Bjork-Shiley		-	Not required	Alive
Kenji M. et al.	52	Male	AVR + MVR	19-mm St. Jude	25-mm St. Jude	-	IABP	Alive
Fisher T. A. et al.	23	Male	AVR (first operation)	St. Jude		-	Not required	Alive
	35	Male	MVR (second operation)		St. Jude	-	Not required	Alive
Katayama Y. et al.	27	Female	AVR + MVR	19-mm St. Jude HP	25-mm St. Jude	-	Not required	Alive
Takashi M. et al.	35	Female	AVR + MVR	16-mm ATS-AP	20-mm ATS-AP	-	Not required	Alive
Rocha R. V. et al.	47	Female	AVR + MVR	19-mm St. Jude	25-mm St. Jude	-	Not required	Alive
Felice T. et al.	30	Male	TAVI	26-mm Sapien XT		-	Not required	Alive
Yohei S. et al.	56	Female	AVR + MVR	Not described	Not described	Yes	Not required	Alive
Kstsukiyo K. et al. (present case)	41	Female	MVR (first operation)		20-mm ATS	-	IABP/VA-ECMO	Alive
Yusuke Y. et al. (present case)	51	Female	TAVI (second operation)	26-mm Sapien XT		-	Not required	Alive
	54	Female	AVR (third operation)	16-mm ATS-AP		Yes	IABP/VA-ECMO	Dead

*AVR* aortic valve replacement, *IABP* intra-aortic balloon pump, *MVR* mitral valve replacement, *TAVI* transcatheter aortic valve implantation, *VA-ECMO* venoarterial extracorporeal membrane oxygenation

The novelty of this study was to firstly report the histological findings of a bioprosthetic valve explanted early following TAVR, providing new insight into the pathophysiology behind the early SVD in patients with mucopolysaccharidosis. Pathohistological examination revealed an accumulation of GAGs on the leaflets of the bioprosthetic valve, which may have been the cause of early SVD (Fig. 1). Pathohistological changes in native aortic valves and annulus also indicate that the primary aortic stenosis may be caused by Scheie syndrome. As there are no previous reports of SVD following TAVI in patients with this entity, we can only speculate that the durability of the implanted biological valve may be affected by accumulated GAGs in the prosthetic valves.

This report is also the first to report a pathological study of coronary artery stenosis in patients with Scheie syndrome. Luminal narrowing of coronary artery due to intimal proliferation and GAGs deposition is well described in Hurler syndrome (severe form of MPS-I) and other types of MPS, but poorly understood in other “non-Hurler” phenotypes of of MPS-I, such as the milder forms like Scheie syndrome and Hurler-Scheie syndrome [6, 7]. There are only two case reports on patients with Scheie syndrome who underwent concomitant CABG with valve surgeries. Our pathohistological findings might suggest that coronary artery could also be affected in patients with the attenuated form of MPS-I (Fig. 4).

As our report, patients with Scheie syndrome often suffer from low output syndrome preserved left ventricular ejection fraction, which is diagnosed of left ventricular diastolic dysfunction. It may be caused by inadequate myocardial protection; however, from pathological autopsy findings, there were not any necrosis or infiltration of inflammatory cells in myocardium. It is also caused by myocardial proliferation due to GAGs deposition and endocardial fibroelastosis [1]. However, in our case, GAGs were not deposited in the left ventricular wall (Fig. 5). A previous report noted that enzyme replacement therapy extinguishes GAGs deposition in the left ventricular wall; however, it does not improve cardiac function[5]. We hypothesize that our patient could be in a similar situation, and diastolic dysfunction was aggravated by cardiac edema due to intraoperative cardioplegic arrest.

In conclusion, our case indicated valve dysfunction due to an accumulation of GAGs not only in native aortic valve but also in bioprosthetic valve, which suggested the importance of valve selection in Scheie syndrome.

## Data Availability

Data sharing is not applicable to this article as no datasets were generated or analyzed during the current study.

## Abbreviations

AVR: Aortic valve replacement
CABG: Coronary artery bypass grafting
GAGs: Glycosaminoglycans
MPS-I: Mucopolysaccharidosis type I
SVD: Structural valve deterioration
TAVI: Transcatheter aortic valve implantation
